# Change in the graphics of journal articles in the life sciences field: analysis of figures and tables in the journal “*Cell*”

**DOI:** 10.1007/s40656-022-00516-9

**Published:** 2022-08-02

**Authors:** Kana Ariga, Manabu Tashiro

**Affiliations:** 1 Division of Cyclotron Nuclear Medicine, Cyclotron and Radioisotope Center, Tohoku University, Sendai, Japan; 2 LAIMAN, Inc., Tokyo, Japan; 3 College of Arts and Sciences, J. F. Oberlin University, Tokyo, Japan

**Keywords:** Diagram, Scientific visualization, Illustration, Figure, Visual culture, Visual representation

## Abstract

The purpose of this study is to examine how trends in the use of images in modern life science journals have changed since the spread of computer-based visual and imaging technology. To this end, a new classification system was constructed to analyze how the graphics of a scientific journal have changed over the years. The focus was on one international peer-reviewed journal in life sciences, *Cell*, which was founded in 1974, whereby 1725 figures and 160 tables from the research articles in *Cell* were sampled. The unit of classification was defined as a graphic and the figures and tables were divided into 5952 graphics. These graphics were further classified into hierarchical categories, and the data in each category were aggregated every five years. The following categories were observed: (1) data graphics, (2) explanation graphics, and (3) hybrid graphics. Data graphics increased by more than sixfold between 1974 and 2014, and some types of data graphics including mechanical reproduction images and bar charts displayed notable changes. The representation of explanatory graphics changed from hand-painted illustrations to diagrams of Bezier-curves. It is suggested that in addition to the development of experimental technologies such as fluorescent microscopy and big data analysis, continuously evolving application software for image creation and researchers’ motivation to convince reviewers and editors have influenced these changes.

## Introduction

In recent science studies, visual cultures in science have been actively discussed, addressing science in social, historical, and philosophical contexts by analyzing various visual representations, including pictures, scientific visualizations, and illustrations. In recent years, approaches such as the social history of science, ethnomethodology, and actor-network theory have become more common, and interest in the visual culture of science has expanded to the interaction and relationship between science and social situations, digital technology and representation practices, and the acceptance and diffusion of visual representation (Burri & Dumit, 2008; Coopman et al., 2014). In these discussions, a trend called “the visual turn” in science has been pointed out by Carusi et al. (2015), among others. Because the use of computer-based images has become widespread, and big data and simulations have grown in complexity, data visualization appears to be an indispensable method for science. In addition, the development of technology for “seeing” objects has been remarkable. Various imaging technologies, such as microscopy, radiography, magnetic resonance imaging (MRI), and live imaging, have been reported (Hentscel, 2014). Thus, the link between science culture and visualization technologies seems to be more pronounced.

It is not just experimental technology that brings about changes in scientific images. Many scholarly journals have shifted from traditional paper media to online publications. Due to the large volume of academic papers being published (Larsen & Ins, 2010), the use of graphical abstracts that intend to help readers grasp the content quickly has become widespread (Editorial of Nature Chemistry, 2011). Most journal publishers have implemented electronic submission systems, and the problems associated with manipulated and falsified digital images have become easily detectable. Digital image processing is easier than ever before, and some journals have established guidelines for digital image processing (Frow, 2014). These changes in media and image tools may be related to the practices of researchers.

The purpose of this study is to examine how trends in the use of visual representations in modern life science journals have changed since the spread of computer-based visual and imaging technologies. The relationship between scientific images, research practices, and experimental technologies in life sciences have been discussed in some fields. In cognitive sciences, the characteristics and context of diagrams have been studied, and the role of diagrams in reasoning has been experimentally verified (Blackwell & Engelhardt, 2002; Hegarty, 2011; Kulpa, 1994; Tversky, 2011). In the philosophy of science, some philosophers have pursued the explanatory role of diagrammatic representations in reasoning and the relationship between images and scientific practice (Abrahamsen et al., 2018; Bechtel & Abrahamsen, 2005; Bechetel et al., 2014; Burnston et al., 2014; Griesemer, 1991; Perini, 2010; Sheredos & Bechtel, 2016, 2017). However, these researchers in cognitive science and the philosophy of science have analyzed diagrams at specific times, and thus far seem not to have attended to changes in visualization practices over time. On the other hand, in the history of science, it has been argued that visual images and representational practices have historically changed. These changes are related to the change in experimental equipment and/or related practices (Griesemer, 2007; Ramussen, 1997; Schickore, 2007), technological developments such as “computer revolution” (Krohn, 1991), social requirements (Hankins, 1999) and changes in thought as well as the epistemic virtue of researchers (Daston & Galison, 2007; Maienschein, 1991). However, few studies have focused on visual representation in scientific journals. It is also not clear what kinds of visual representations are used and to what extent.

This study focuses on the types and quantitative changes in visual representations in scientific journals and discusses the factors behind these changes. How should these representations be classified to clarify the changing relationship with the spread of visual and imaging technologies? Several studies have classified the images used in science (Gross & Harmon, 2014; Hullman & Bach, 2018; Myers, 1990; Pauwels, 2006; Perini, 2010). For example, Myers (1990) analyzed pictures in the textbook, “Sociobiology: The New Synthesis” published in 1975 and distinguished five categories based on the degree of detail: (1) photographs, (2) drawings, (3) maps, (4) graphs, models, and tables, and (5) imaginary figures. However, this classification does not cover all images in scientific journals. Pauwels (2006) classified visual representations according to their referents: material/physical, and mental/conceptual. This classification is useful, but too abstract to observe the influence of imaging technologies. Hullman and Bach (2018) collected 54 graphical abstracts and categorized their design patterns into four categories: (1) layout, (2) time description, (3) text usage, and (4) expression genre. With such a classification, focusing mainly on visual design, it is difficult to discuss the influence of technological changes.

In this study, we designed a new classification system for scientific representations, retrospectively, in a manner that foregrounds experimental and representational technologies. Visual representations in journal articles were categorized accordingly, and the changes in the numbers of each type of representation were analyzed. We observed that more data images representing data visually have been placed in research articles, and the representation styles of the explanatory diagrams have changed over time. We suggest that, in addition to the development of experimental technologies noted in previous studies, image creation software and researchers’ motivation to convince reviewers and editors of the value of their work have influenced the practice of visually representing data and ideas with figures, including images, photographs, graphs, tables, drawings, and other depictions.

## Research method

### About the journal “*Cell*”

In this study, “Figures” and “Tables”^1^ in *Cell,* which is an international peer-reviewed scientific journal published by Cell Press (Elsevier) were analyzed. *Cell* was established in 1974 and covers areas of experimental biology, including but not limited to cell biology, molecular biology, neuroscience, immunology, virology and microbiology, cancer, human genetics, systems biology, signaling, disease mechanisms, and therapeutics.^2^

**Fig. 1:**
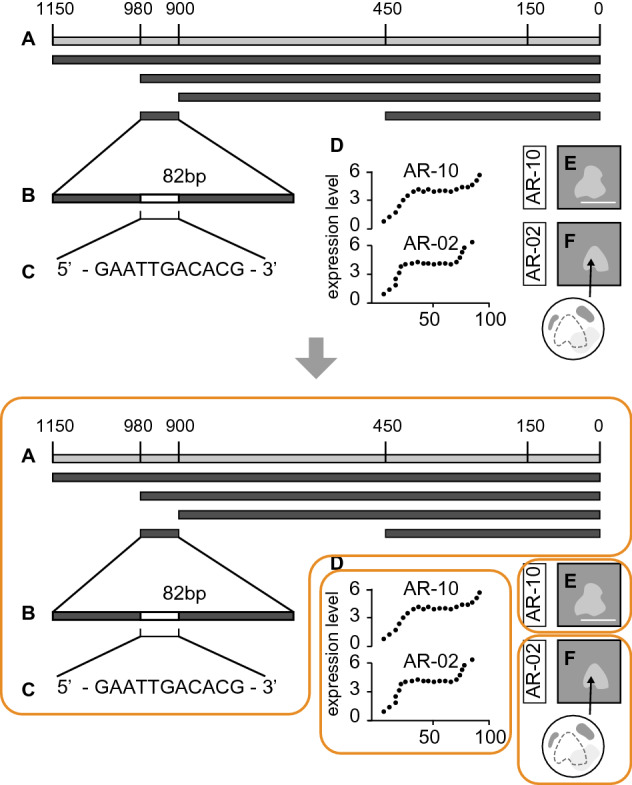
A method of separating graphics with a single drawing line. In this case (an imaginary figure), four graphics are counted

**Fig. 2:**
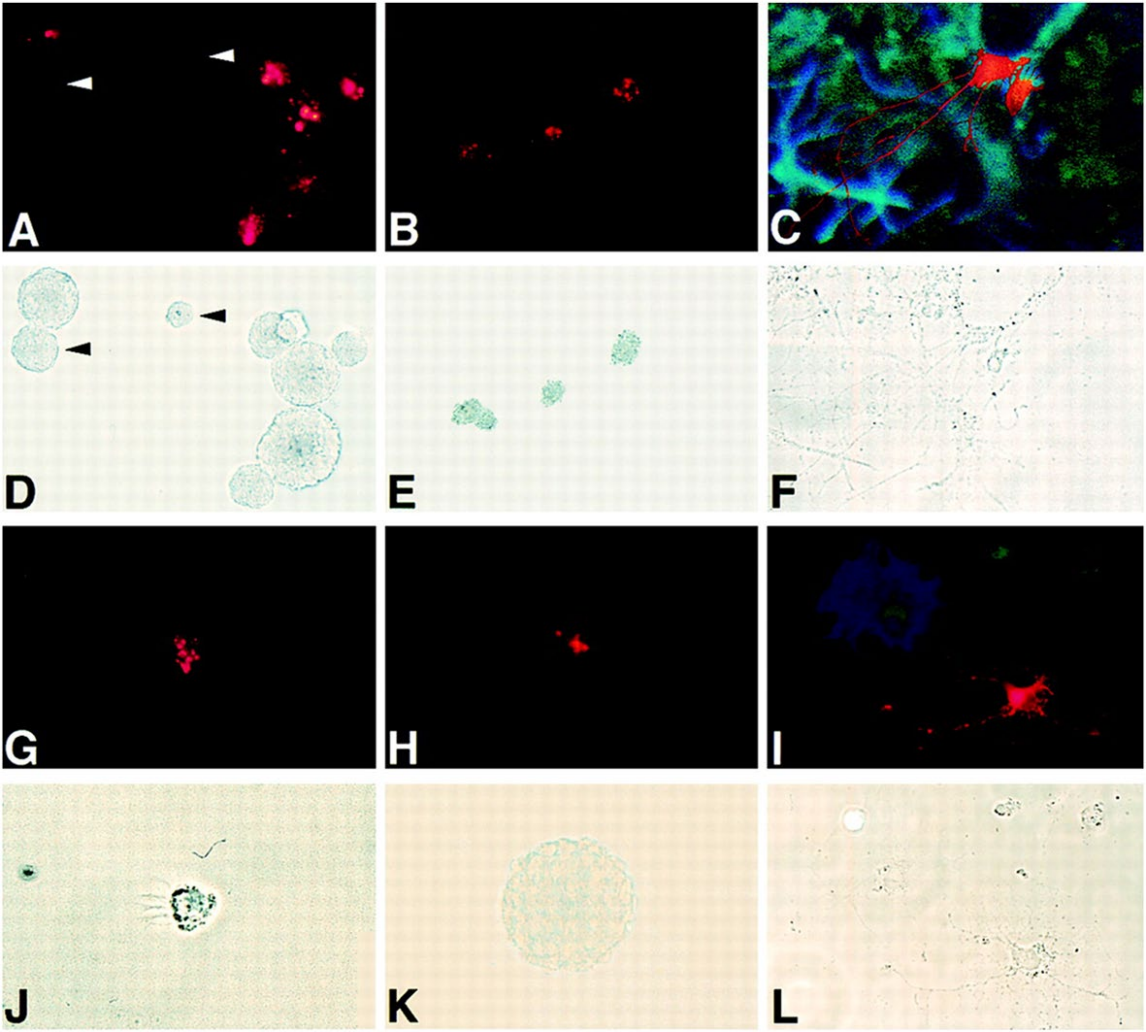
Examples of mechanical reproduction images (Reprinted from Johansson et al., 1999, Fig. 2A–L with permission from Elsevier)

The first reason for choosing this journal is that *Cell* is one of the top journals in the field of life sciences. According to journal citation reports, the journal, in 2020, had an impact factor of 41.582. It is one of the most renowned journals in all research fields. Second, the journal was launched in 1974. The emergence of personal computers occurred in the late 1970s and hence it is appropriate to explore the changes that occurred after the spread of computer-based visual and imaging technologies.

### Definition of graphical units

The definition of image units is important for classification. In journal articles, a single “Figure” may contain several elements (including graphs, photographs, and pictures). Some “Figures” have alphabets assigned to each graphic element, while others do not. In such a case, the “Figure” cannot be employed as a classification unit. Therefore, it is necessary to define the classification unit in this study before the classification can be performed. In this study, the elements constituting classification units are called “graphics” to distinguish them from “Figures” and “Tables” and are defined as follows.

A single graphic is that which can be visually separated, can make sense even if it is independent, and can be surrounded by a single drawing line (Fig. 1) in “Figures” and “Tables” without captions. If meaningless symbols are generated outside one graphic and make more sense when combined with the graphic, a line is redrawn and counted as one graphic.

In the case of more than one graphic element sharing legends or titles, the elements are counted as different graphics if parts, excluding the legends or titles, can be separated by the method described above, except for the following cases. If elements sharing legends or titles are classified in band and blotting images and the results are apparently continuous in the same type of experiment, they are combined into one.

When two or more graphic elements explicitly share a vertical or horizontal axis, the shared elements are counted as one graphic. If two or more independent graphic elements have the same proportions, are aligned, share indication symbols, such as lengths and numbers, and are comparable to each other, they are counted as one graphic. Moreover, if two or more graphic elements are arranged in parallel without sharing any guide or indication symbols, the elements are counted as separate graphics, even if they can be compared to each other. This is because in many cases, it is unclear whether they can be compared. On the other hand, in the case of stationary images extracted from one movie image being continuously arranged and displayed, the continuous images are collectively counted as one graphic.

### Analytical method

In this study, a graphic classification framework was created for the research articles in the journal. The number of graphics in each category was aggregated every five years to examine the trends in graphics. The research method was as follows.

First, a database was built for all the articles in the journal, including information about authors, titles, years, volumes, issues, first pages, last pages, and article types. Only research articles were selected for the classification. Other articles, such as review articles and letters, were removed from the list because they differ in the way images are used. Second, a classification framework was constructed according to the following procedure: (1) The “Figures” and “Tables” of the first five research articles of the year were collected, every five years from 1974 to 2014, and divided into the graphic units described in Sect. 2.3. (2) The “Figures” and “Tables” were roughly categorized according to the classification of Myers (1990) taking note of items that did not fall into it. (3) All “Figures” and “Tables” were printed, labeled, grouped and arranged into larger groups using the KJ method^3^(Scupin, 1997). (4) The groups were examined for duplication or inconsistency, and the classification was modified as needed to provisionally complete the classification.^4^ (5) To examine whether this classification framework could be applied to other graphics, “Figures” and “Tables” for the next 15 articles were additionally classified every five years and the framework was modified accordingly. The categories were arranged into a hierarchy in this classification process. The family groups were merged into three categories. (6) In recent years, a great deal of material has been included in the supplementary information. In order to check the trends of graphics in the supplementary information, the “Figures” and “Tables” in the supplementary information of the articles were also classified. Eventually, 1,725 “Figures,” 160 “Tables,” and 5,952 graphics in the main bodies of the articles, and 296 “Figures,” 78 “Tables,” and 2,150 graphics in the supplemental information of the articles (Table 1) were categorized.

**Table 1:** Numbers of “Figures”, “Tables” and graphics that authors analyzed

	Preliminary classification			Additional classification			Total in main bodies			Total in supplemental information		
	Figures	Tables	Graphics	Figures	Tables	Graphics	Figures	Tables	Graphics	Figures	Tables	Graphics
1974	20	14	56	68	26	142	88	40	198	–	–	–
1979	32	5	110	103	19	299	135	24	409	–	–	–
1984	37	3	108	95	12	247	132	15	355	–	–	–
1989	28	8	69	96	22	239	124	30	308	–	–	–
1994	39	5	168	111	17	498	150	22	666	–	–	–
1999	33	3	165	90	13	360	123	16	525	0	2	2
2004	34	2	205	94	10	774	128	12	979	13	12	119
2009	35	0	264	103	1	1057	138	1	1321	169	28	1050
2014	34	0	288	103	0	903	137	0	1191	114	36	979
Total	292	40	1433	862	120	4519	1725	160	5952	296	78	2150

Third, the data were summarized for each publication year. For the statistical analyses of the trend of categories, data were examined using one-way ANOVA, followed by Tukey's post-hoc tests. The details of the graphics that account for a large percentage of the categories were investigated to determine the usage trends that changed significantly and analyze the factors that influenced the trends.

## Result and discussion

### Construction of classification system

As discussed in the Introduction, the classification categories provided in previous works do not suffice to discuss the influence of new technologies on graphics. As a result of the classification focused on experimental and representational technologies, we divided the graphics into three main groups, including (A) data graphics, (B) explanation graphics, and (C) hybrid graphics. Data graphics represented numerical facts (Friendly & Wainer, 2021) and were usually generated mechanically. Explanation graphics were designed and drawn by hand through human epistemic activities. Of note, the roles of data graphics and explanation graphics in scientific arguments differ significantly. Scientists use data to infer facts about phenomena (Bogen & Woodward, 1988) and thus, data serves as evidence. Friendly and Wainer (2021) argued that data graphics which visually display numeric facts support arguments as evidence. In contrast, explanation graphics including scientific diagrams and illustrations play an important role in explaining hypotheses, interpretations, and ideas (Abrahamsen et al., 2018; Bechtel & Abrahamsen, 2005; Tversky, 2011). Data graphics seem to be more closely related to the epistemic virtue of mechanical objectivity (Daston & Galison, 2007) than explanation graphics, owing to the interest in their reliability as evidence. In comparison, explanation graphics seem to emphasize the role of understanding and communication more. Hence these categories not only differ in terms of their generation technologies, but also perform different functions. We considered them as a useful classification to investigate the characteristics of graphics used in scientific literature. The characteristics of these three categories and the subcategories in (A) and (B) are described below.

(A) Data graphics

We define a data graphic as a representation of data. Most data graphics are generated mechanically. The design patterns and ways to visualize graphics depend on the machines employed and the scientific conventions adopted. The following groups were included in this category:

(A1) Mechanical reproduction images

A mechanical reproduction image is an image recorded by instruments for automatically producing unmediated images (Lynch, 1991). It represents the appearance of that object. This is presented as evidence of reality (Perini, 2012) but is not a simple representation of nature. It consists of carefully orchestrated scenes (Chadarevian, 2017; Lynch & Woolgar, 1990). Examples of these images include micrographs, fluorescence micrographs, electron micrographs, differential interference contrast micrographs, and optical images, among others. (See Fig. 2 and the following figures for examples: Rohrschneider, 1979, Fig. 2; Guo, 2014, Fig. 3B.)

**Fig. 3:**
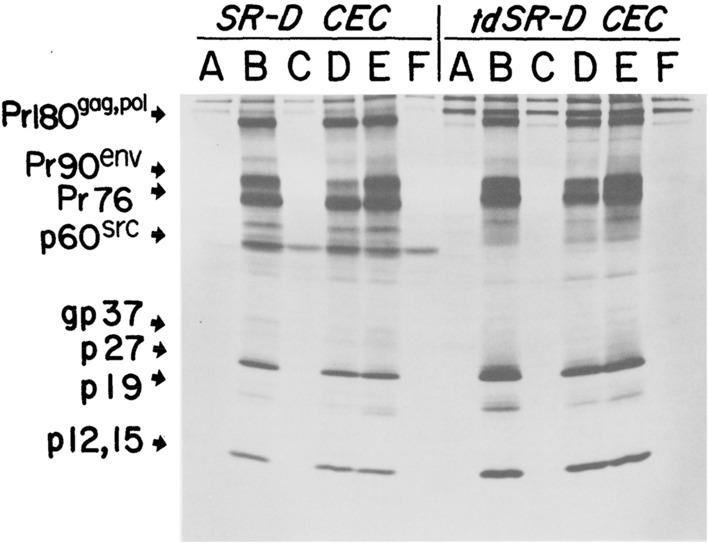
An example of a band and blotting image (Reprinted from Rohrschneider, 1979, Fig. 1 with permission from Elsevier)

(A2) Band and blotting images

We define a band and blotting image as a scanned image of electrophoresis, chromatography, and various types of blotting to detect and analyze target particles, such as nucleic acids and proteins. These images are often the subject of scientific misconduct (image manipulation) (Frow, 2014). Rheinberger (2011) referred to the chromatography of radioactive tracing as a characteristic example of a “tracer” in molecular biology and argued that its visualization meant transformations from “traces” to “data.” These images have a graph-like property, that is, they have coordinates and properties based on the position of the spot on the graph. They can also be regarded as a case in which the subject of the study is mathematically transformed (Lynch, 1990). Examples of these images include electrophoresis photographs, southern blotting images, northern blotting images, and western blotting images (see Fig. 3 and the following figures for examples: Willis et al., 1999, Fig. 1b; Zhang et al., 2009, Fig. 3c.)

(A3) Mechanical data visualization images

**Fig. 4:**
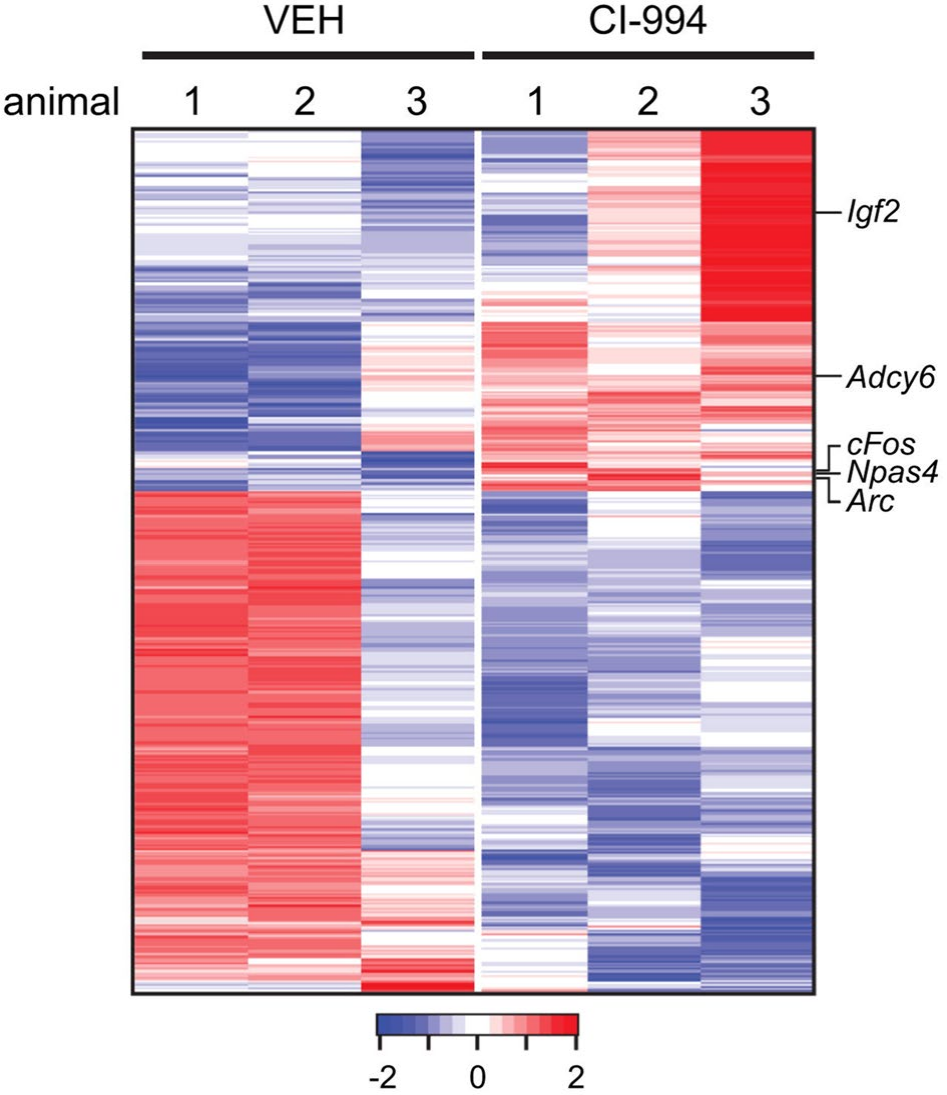
An example of a heat map showing gene expression (Reprinted from Gräff et al., 2014, Fig. 5D with permission from Elsevier)

**Fig. 5:**
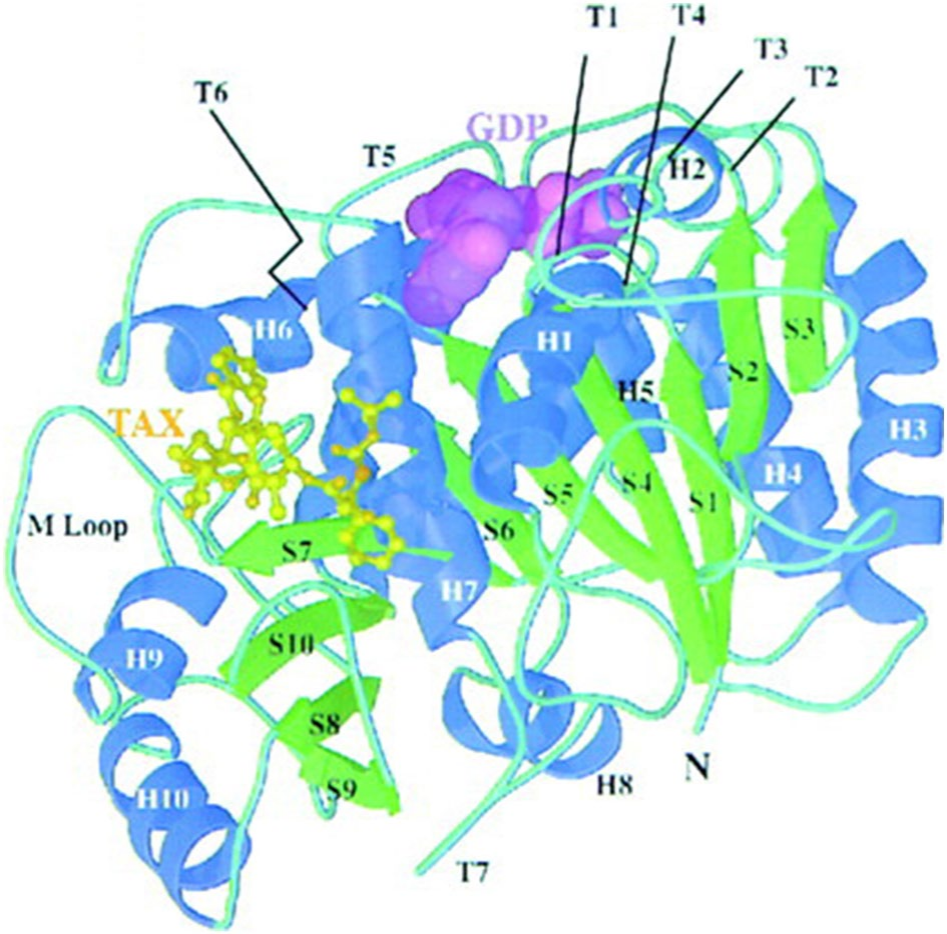
An example of a mechanical data visualization image (Reprinted from Nogales et al., 1999, Fig. 1a with permission from Elsevier)

We define mechanical data visualization images as images of data that were mechanically generated in a format dependent on the device and software used (excluding other groups of data graphics). Some graphics in this category, such as line and bar graphs, can be hand-drawn. Hankins (1999) described that the advantage of graphs is that they provide a **“**bird's eye view” that integrates an enormous amount of data into a single diagram. These graphs allow us to observe regularities and anomalies in the data that are difficult to detect in tables. For example, a heat map showing gene expression is a collection of numerous numerical data, and the patterns of expression levels can be detected at a glance by the difference in colors (Fig. 4). Examples of these images include sequence logos, three-dimensional computer graphics (3DCGs), heat maps, network diagrams, line graphs, and bar graphs (see Fig. 5 and the following figures for examples: Baker et al., 1974, Fig. 1; Gräff et al., 2014, Fig. 5D.)

(A4) Tables

**Fig. 6:**
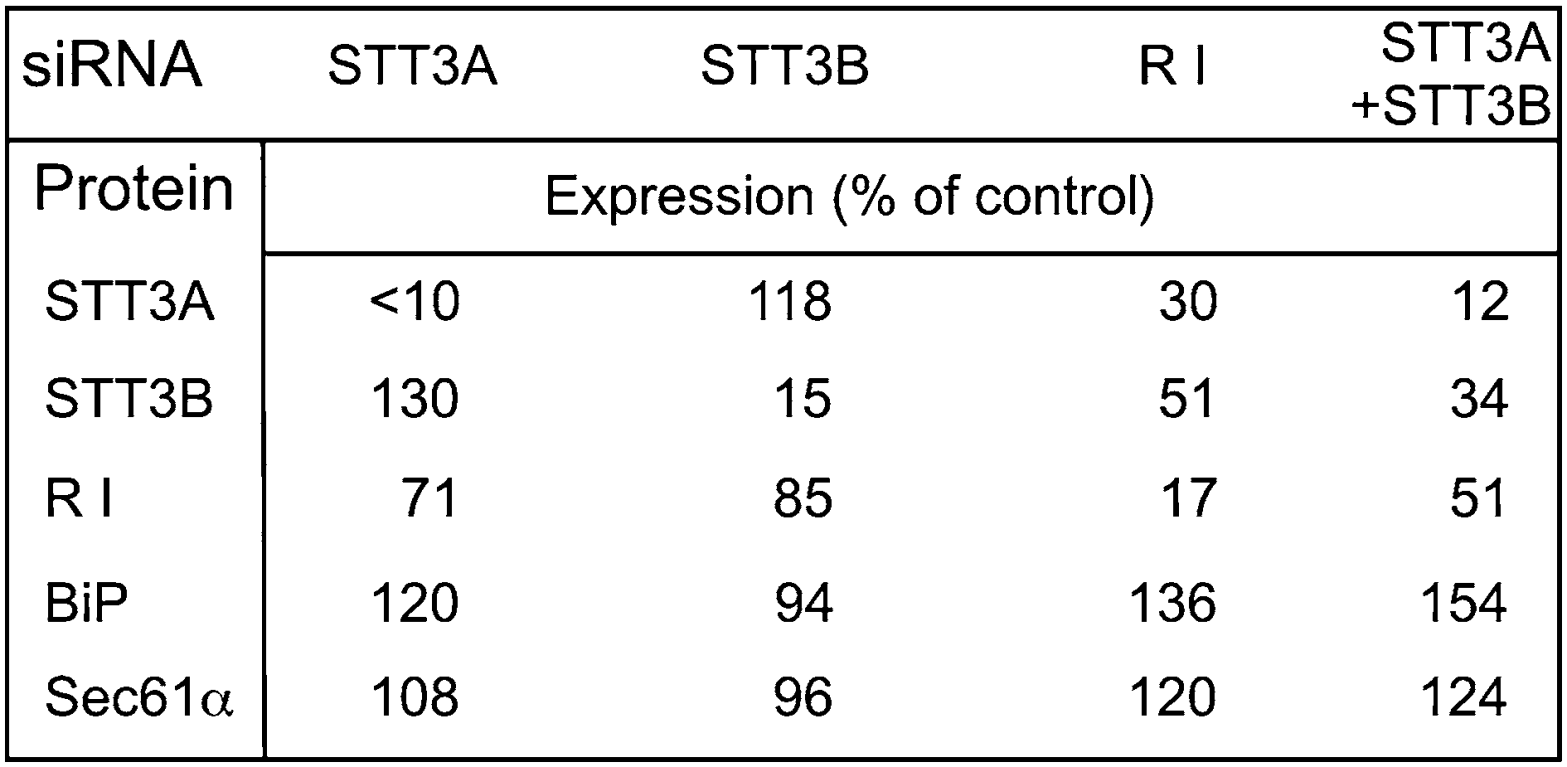
An example of a table (Reprinted from Ruiz-Canada et al., 2009, Fig. 1 with permission from Elsevier)

A table is a two-dimensional arrangement of data (such as numerical values or text characters) in vertical and horizontal columns (Gross & Harmon, 2014). The primary purposes of table are data presentation and retrieval. It is divided into straight lines (which may or may not be visible) and often includes letters, symbols, and simple figures. Tables are created either manually or mechanically. They may be presented as both a “Table” and a “Figure.” Tables work best for conveying precise values and are processed differently from bar and line graphs (Gross & Harmon, 2014). (See Fig. 6 and the following figures for examples: Drews et al., 1974, Table 1; Willis et al., 1999, Table 1).

(A5) Sequences

**Fig. 7:**
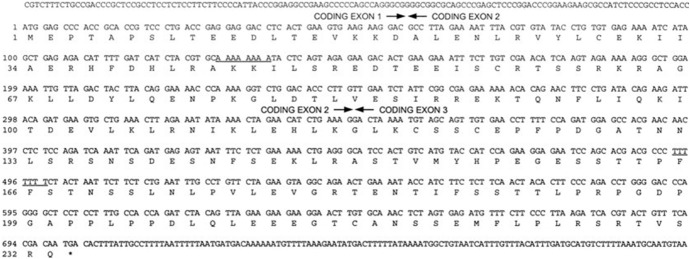
An example of a sequence graphic (Reprinted from Willis et al., 1999, Fig. 2a with permission from Elsevier)

We define a sequence graphic as a sequence of DNA, RNA, or protein strands indicated only by abbreviations (letters). These are presented in two ways: as text and as “Figures.” In this study, only those displayed as “Figures” were considered for classification. (See Fig. 7 and the following figures for examples: Rommelaere et al., 1979, Fig. 6; Zhang et al., 2009, Fig. 3b.)

(A6) Hybrids of data graphics

**Fig. 8:**
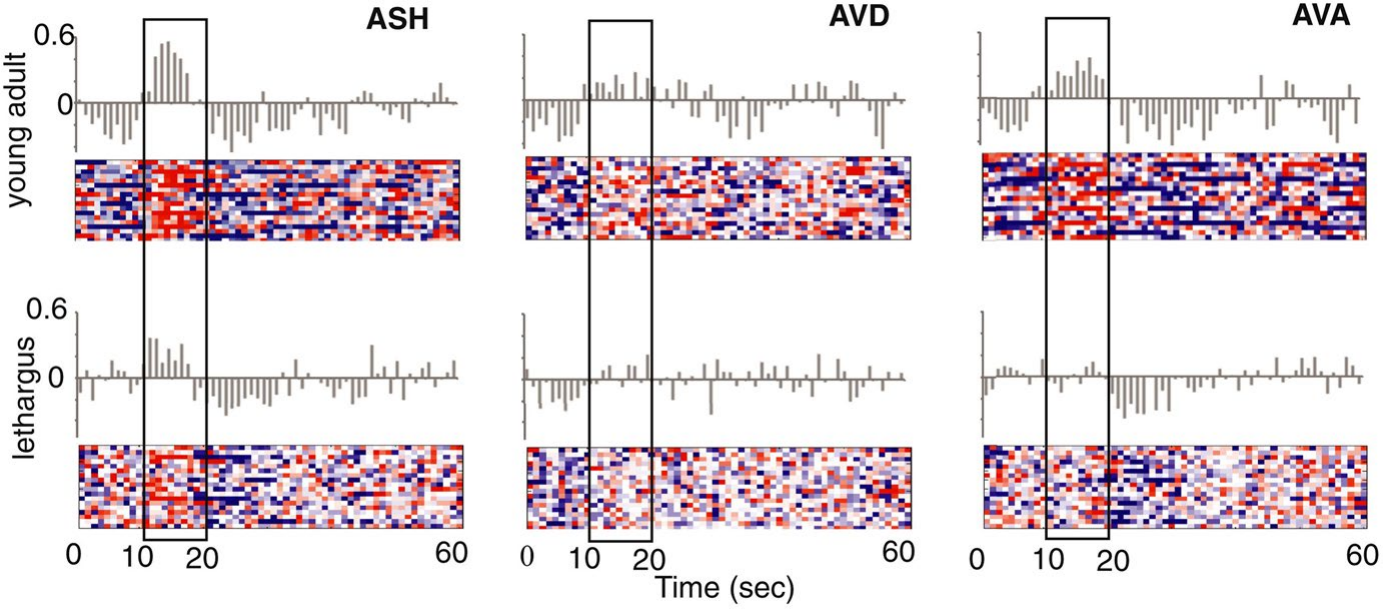
Examples of hybrids of data graphics (Reprinted from Cho & Sternberg, 2014, Fig. 2C with permission from Elsevier)

We define hybrid data graphics as combinations of two or more types. Various graphics can be combined into a single graphic, such as mechanical data visualization images, band and blotting images, and tables. (See Fig. 8 and the following figures for examples: Chafouleas et al., 1984, Fig. 5; Yuzhakov et al., 1999, Fig. 4E (the lower part).)

(B) Explanation graphics

We define an explanation graphic as an illustration or diagrams designed and drawn by hand (including via software) through human epistemic activities such as interpretation and knowledge. They do not comprise data such as numerical values. Diagrams, which play an important role in inference, also fall into this category (Abrahamsen et al., 2018; Bechtel & Abrahamsen, 2005). Diagrams provide effective control of the reasoning process (Kulpa, 1994). Hegarty et al. (1991) and Hegarty (2011) classified diagrams into three categories: iconic diagrams, schematic diagrams, and charts and graphs. Iconic diagrams and schematic diagrams are categorized under explanation graphics in this study. Some illustrations display the structures, while others illustrate functionality (e.g., movement, cause, and time) (Heiser & Tversky, 2006; Tufte, 2001). While illustrations of form and appearance correspond easily to data representations, such as mechanical representation images, relational diagrams correspond to meaning. As these graphics seem to play different roles in scientific debate, they were categorized separately. Although illustrations could be further categorized, as in other studies (e.g., Hullman & Bach, 2018), they were not categorized in detail here because the small number of counts in each category would make it difficult to observe a trend.

Scientific illustrations are considered to be theory-laden (for example, Daston & Galison, 2007; Ford, 1993; Hentschel, 2014; Kight, 1985; Rudwik, 1976; Toppwer, 1996). Since an explanation graphic explains the concepts or idea, accessing the knowledge and acquiring a sense for the values of researchers is easier than in a data graphic which is a mechanical visualization of the numerical values. Of course, some data graphics also reflect researchers’ epistemic virtues, such as objectivity (Daston & Galison, 2007), but we think that the researcher’s personal sense of values has a stronger influence on the illustrations drawn by hand.

(B1) Illustrations of form and appearance

**Fig. 9:**
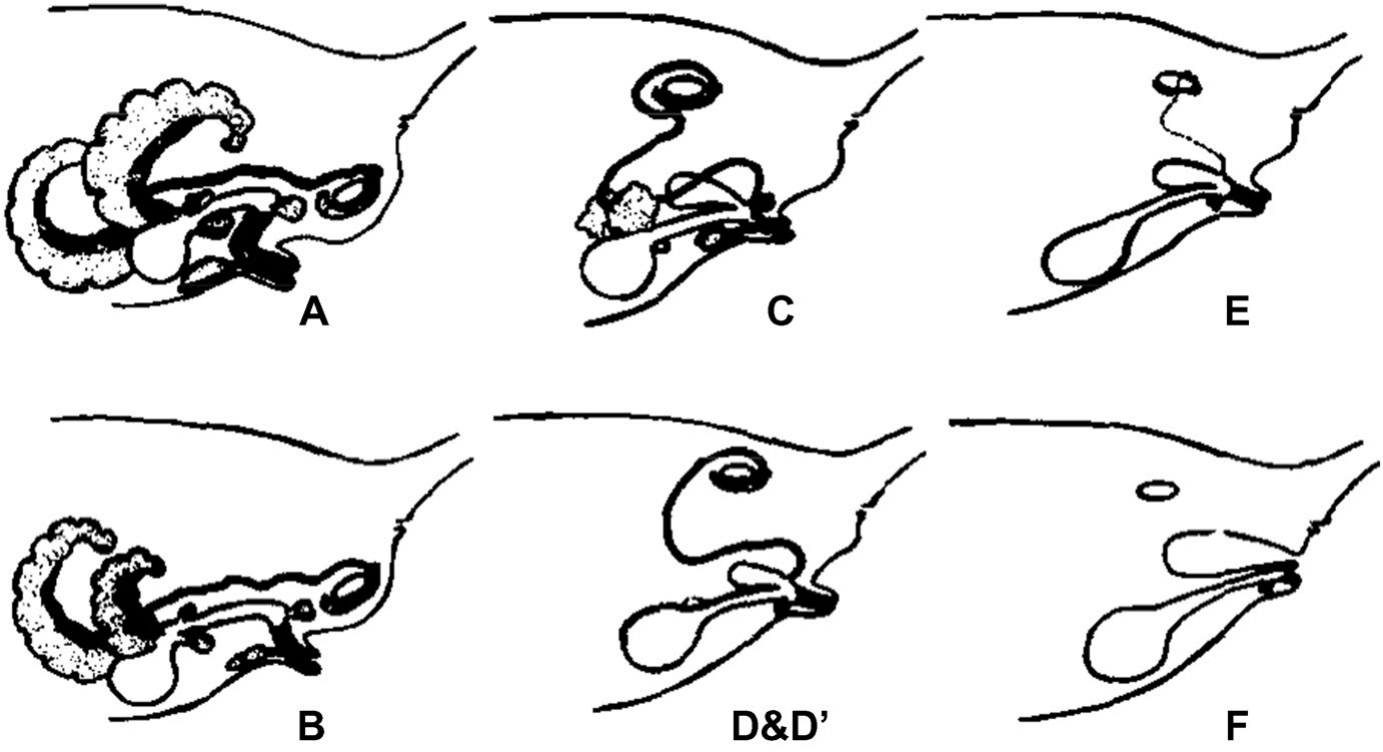
Examples of illustrations of form and appearance (Reprinted from Drews et al., 1974, Fig. 3 with permission from Elsevier)

We define an illustration of form and appearance as an illustration showing the two- or three-dimensional structures of referents. The mechanical reproduction image (A1) also shows the form and appearance of referents. The difference between them is that the former is drawn by hand and the latter is mechanically recorded. This category includes an illustration associated with a photograph, described by Lynch as a “directional” relationship between paired representations (Lynch, 1990). Researchers extract and emphasize important information from the referents in the illustrations. Unlike images in biology textbooks (Myers, 1990; Perini, 2012), there are few real or detailed descriptive expressions in journal articles. Illustrations in this category are often represented as schematic and compositional diagrams (Perini, 2012). Although resemblance is not an explanatory key for visual representation in scientific models (Perini, 2010), illustrations of form and appearance provide more concrete images than relational diagrams do. Some maps and diagrams seem to represent the appearances of referents but focus on presenting positional relationships rather than forms. Illustrations, such as these that do not explain appearances are included in (B2) relational diagrams. (See Fig. 9, and the following figures for examples: England et al., 1999, Fig. 1a; Snaidero et al., 2014, Fig. 7A–D.)

(B2) Relational diagrams

**Fig. 10:**
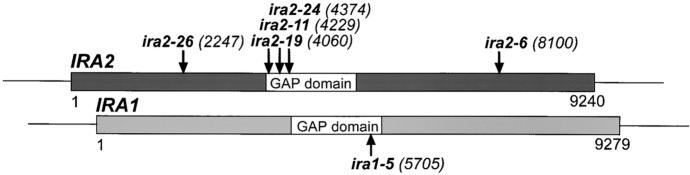
An example of a relational diagram (Reprinted from Halme et al., 2004, Fig. 2B with permission from Elsevier)

We define a relational diagram as a diagram describing the relationships between two or more concepts or items, such as causality, position, time series, classification, and effects, in addition to properties and concepts such as states and conditions. These include graphical signs as visual elements, such as Venn diagrams, comb diagrams, phylogenetic trees, chemical structural formulas, maps, and sequence diagrams (DNA and RNA sequences explained using figures). (See Fig. 10 and the following figures for examples: Bard et al., 1974, Fig. 2; Yang et al., 2014, Fig. 5G.).

(B3) Hybrids of explanation graphics

**Fig. 11:**
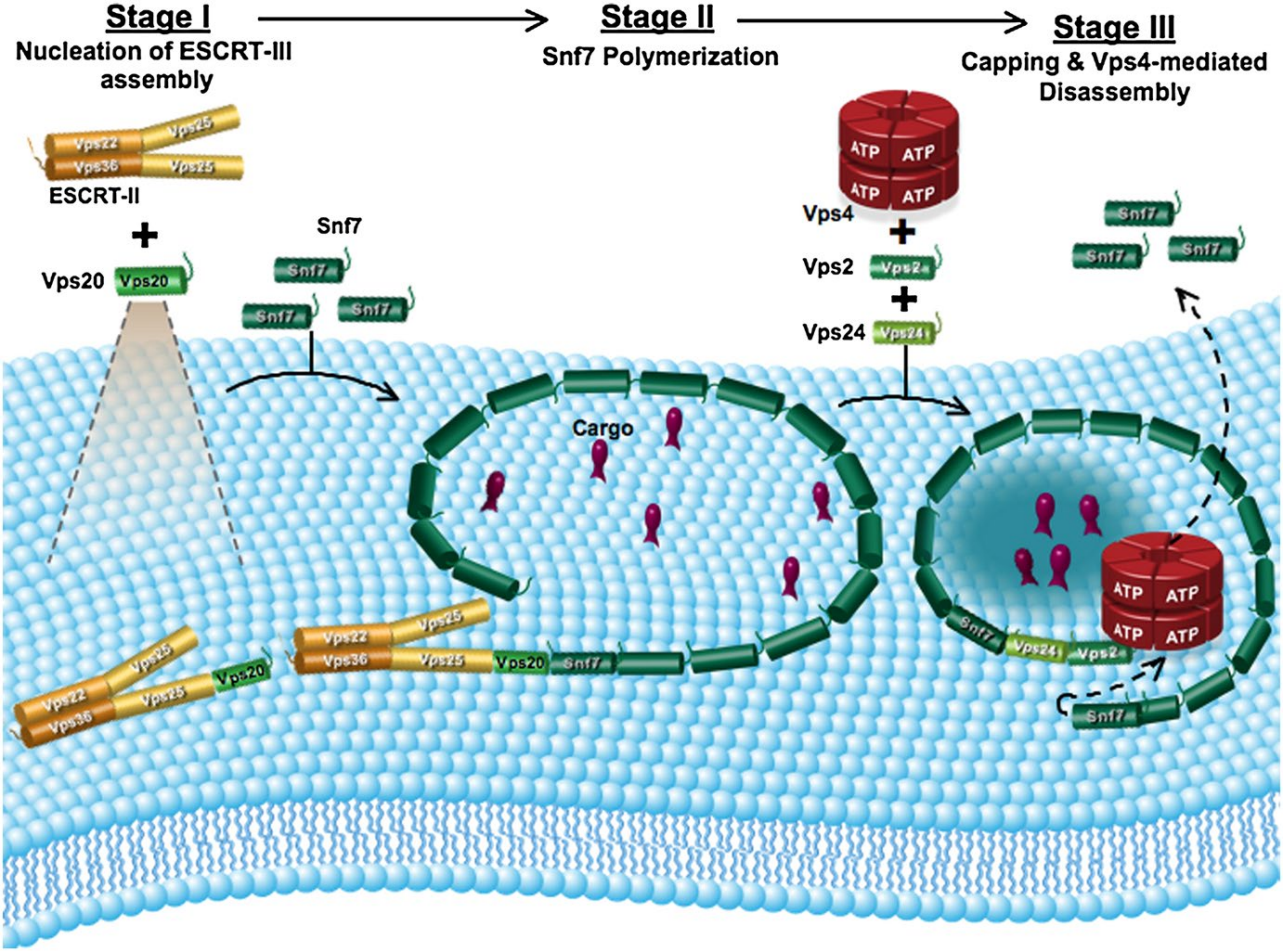
An example of a hybrid of explanation graphics (Reprinted from Saksena et al., 2009, Fig. 7 with permission from Elsevier)

We define hybrid explanation graphics as a combination of illustrations of form and appearance (B1) and relational diagrams (B2). Signs or elements that have a schematic shape and represent objects such as molecules, cells, organisms, experimental machinery and instruments are often connected by arrows or lines to indicate the relationship between them. Most hybrid of explanation graphics were what Abrahamsen et al. called mechanism diagrams, which were composed of different kinds of elements called glyphs such as shapes, lines and arrows, colors, text, iconic symbols, graphic symbols, and embedded data graphs (Abrahamsen et al., 2018). These schematic diagrams have been often used in recent biology, partly because too much detail in pictorial images overwhelms both the audience and research analysis (Lynch, 1991; Maienschein, 1991). (See Fig. 11 and the following figures for examples: Goldenberg & Raskas, 1979, Fig. 9; Johansson et al., 1999, Fig. 1a.)

(C) Hybrid graphics

A hybrid graphic combines (A) data graphics and (B) explanation graphics. Some studies have also acknowledged the existence of hybrids. Gross and Harmon (2014) pointed out that some representations are hybrids of different types of graphics. They pointed to the Coull's trellis diagram as an example as it includes both tabular and graphic representations of the distribution. Abrahamsen et al. (2018) also showed that diagrams can be created by combining different types of elements. They pointed out that data graphics are occasionally integrated into a mechanism diagram.

**Fig. 12:**
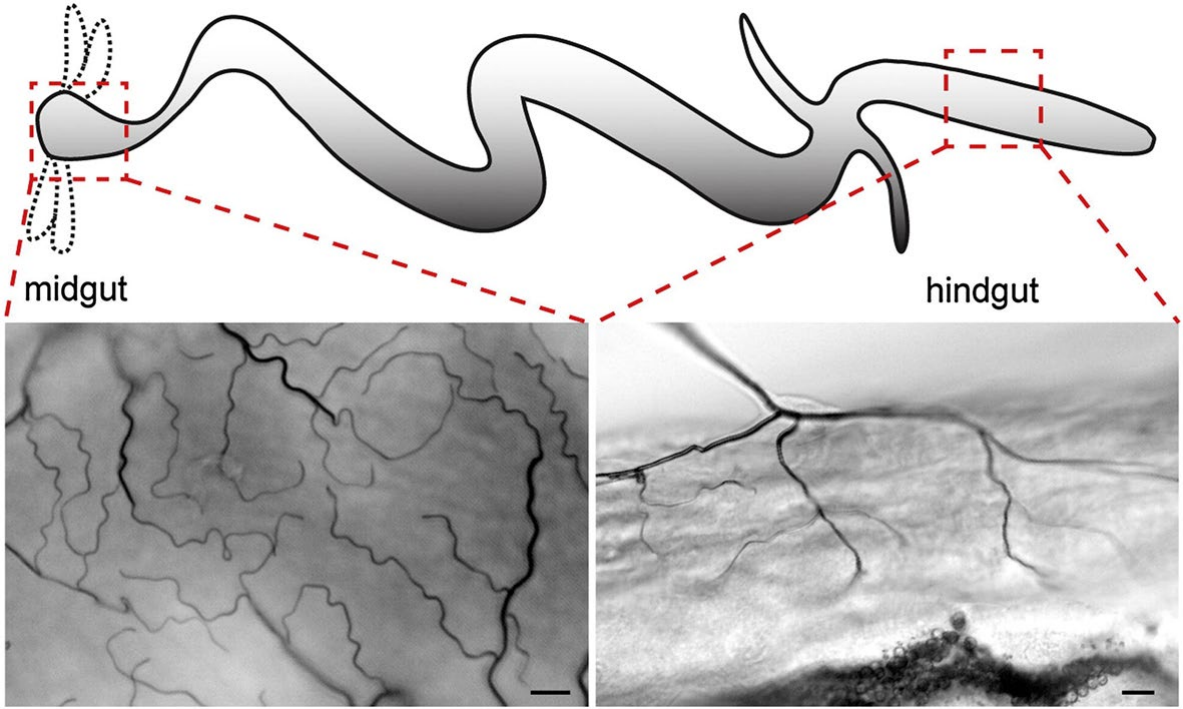
An example of an explanation graphic incorporated in a data graphic (Reprinted from Linneweber et al., 2014, Fig. 2, licensed under Creative Commons Attribution 3.0 Unported License (CC BY 3.0))

**Fig. 13:**
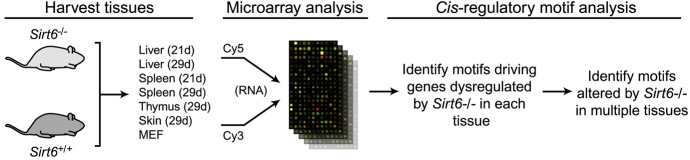
An example of a data graphic inserted as an element of an explanation graphic. In this case, the image of the microarray is actually captured as an icon of the methodology rather than as data to be read (Reprinted from Kawahara et al., 2009, Fig. 6 with permission from Elsevier)

**Fig. 14:**
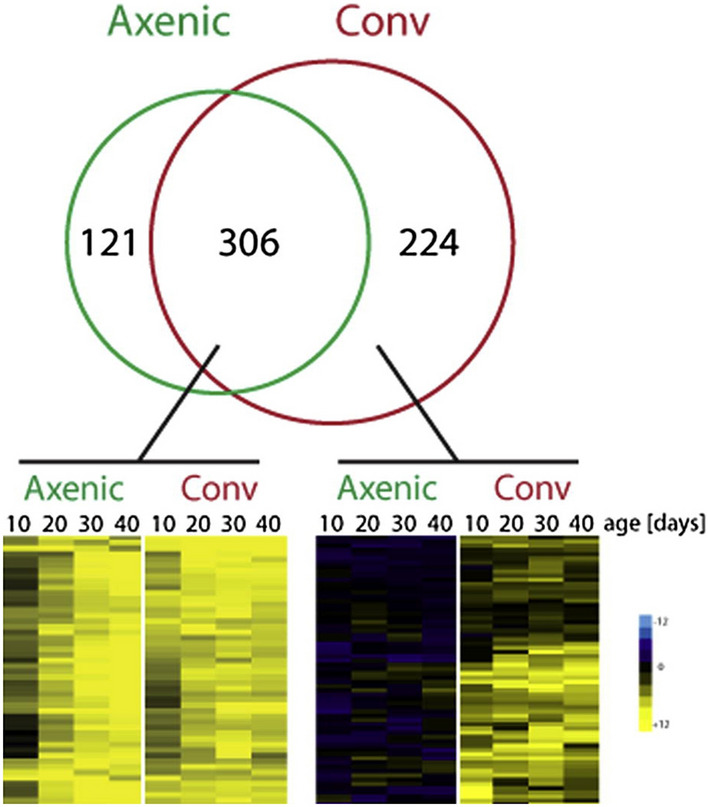
An example of a hybrid graphic (Reprinted from Guo et al., 2014, Fig. 1D with permission from Elsevier)

Hybrid graphics consist of two types: explanation graphics to explain the data incorporated in data graphics (Fig. 12) and data graphics inserted as elements of explanation graphics (Fig. 13). In this research, most hybrid graphics are classified into data graphics or explanation graphics. However, it is known that in some instances, often seen in graphical abstracts, data graphics that require the data to be read are incorporated in an explanatory diagram (for example, the graphical abstracts in the following articles: Beck et al., 2019; Bergmann et al., 2015; Tagliabracci et al., 2015). Graphical abstracts are pictorial and visual summaries of the main findings of articles,^5^ often seen in online scientific journals, and are deeply related to the figures in research articles. These graphics can be both data and explanatory type (see Fig. 14, and the following figures for examples of hybrid graphics: Chapados et al., 2004, Table1; Perissi et al., 2004, Fig. 2F).

### Results of counting and overall trend

**Fig. 15:**
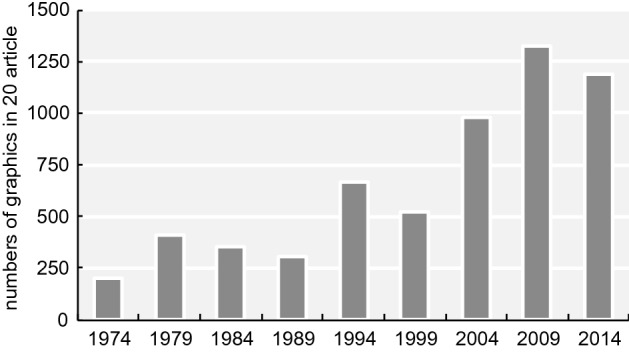
Change in the total numbers of graphics in 20 articles measured since 1974

**Fig. 16:**
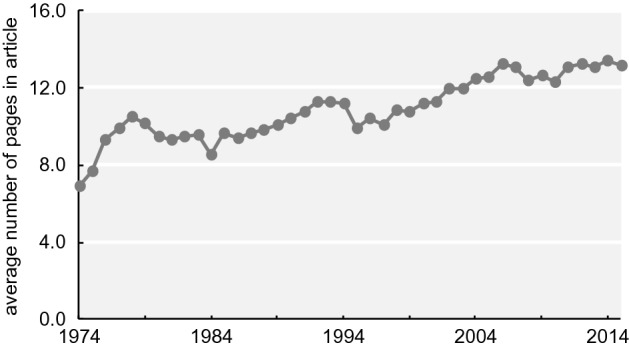
Change in average number of pages per article. These numbers were acquired from the database

The total numbers of graphics in each category for the main articles are shown in Table 2 while Table 3 shows the totals for the supplemental information.^6^ The number of graphics per article increased by 6.7 times from 1974 to 2009 and 6.0 times from 1974 to 2014 (Fig. 15,^7^). There were statistically significant differences between 1974 and 2009 and between 1974 and 2014; specifically, the p-values were less than 0.001 (Table 4). Although the number of “Figures” has been no more than seven (or eight) since 1993, the number of graphics has increased. According to the journal information for contributors, the total character count of an article has been under 55,000 since 1995. On the other hand, the average number of pages per article increased from 6.94 in 1974 to 13.17 in 2014 (Fig. 16; these numbers were obtained from the database). These results suggest that the increase in the number of pages since 1995 is thought to be largely affected by the increase in the number of graphics areas and that researchers in recent years have incorporated several graphics into one “Figure.”

**Table 2:** Classification results of graphics in each category

	1974	1979	1984	1989	1994	1999	2004	2009	2014
Data graphics (total)	183	378	279	252	593	444	863	1196	1105
Mechanical reproductions	26	191	100	31	324	124	462	638	427
Band images	4	83	78	100	124	48	107	174	48
Mechanical data visualizations	112	71	65	73	112	234	219	341	551
Tables	40	24	16	32	25	15	15	5	11
Sequences	0	7	13	14	4	12	7	8	7
Data hybrid	1	2	7	2	4	11	53	30	61
Explanation graphics (total)	12	30	57	45	68	70	87	96	60
Illustrations of form and appearance	7	26	26	37	45	42	56	58	26
Relational diagrams	1	1	3	1	4	1	2	3	14
Hybrids of explanation graphics	4	3	28	7	19	27	29	35	20
Hybrid graphics (total)	3	1	19	11	5	11	29	29	26
Graphics total	198	409	355	308	666	525	979	1321	1191

**Table 3:** Classification results of graphics in supplemental information in each category

	1974	1979	1984	1989	1994	1999	2004	2009	2014
Data graphics (total)	–	–	–	–	–	2	101	1009	910
Mechanical reproductions	–	–	–	–	–	0	79	522	320
Band images	–	–	–	–	–	0	6	114	45
Mechanical data visualizations	–	–	–	–	–	0	4	306	471
Tables	–	–	–	–	–	2	12	48	38
Sequences	–	–	–	–	–	0	0	12	0
Data hybrid	–	–	–	–	–	0	0	7	36
Explanation graphics (total)	–	–	–	–	–	0	12	35	56
Illustrations of form and appearance	–	–	–	–	–	0	0	20	28
Relational diagrams	–	–	–	–	–	0	12	12	22
Hybrids of explanation graphics	–	–	–	–	–	0	0	3	6
Hybrid graphics (total)	–	–	–	–	–	0	6	6	13
Graphics total	–	–	–	–	–	2	119	1050	979

**Table 4:** Results of post hoc test of graphics (p values)

	1974	1979	1984	1989	1994	1999	2004	2009	2014
1974									
1979	0.632								
1984	0.898	1.000							
1989	0.987	0.993	1.000						
1994	0.002**	0.358	0.134	0.045*					
1999	0.094	0.982	0.849	0.595	0.943				
2004	0.000**	0.000**	0.000**	0.000**	0.129	0.003**			
2009	0.000**	0.000**	0.000**	0.000**	0.000**	0.000**	0.066		
2014	0.000**	0.000**	0.000**	0.000**	0.000**	0.000**	0.626	0.964	

**p < 0.01, *p < 0.05

### Trend by category

**Fig. 17:**
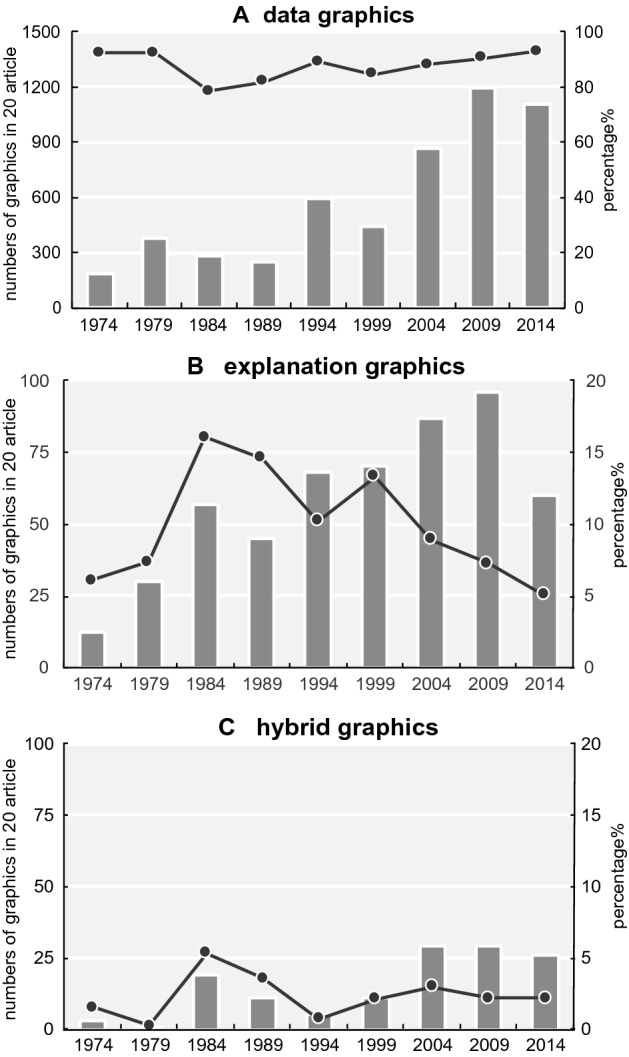
Changes in the numbers and ratio of the three categories (a: data graphics, b: explanation graphics, c: hybrid graphics). The bar graph depicts real numbers and the line graph depicts percentages of for total graphics of each year

The changes in the three main categories are presented in Fig. 17 (a: data graphics, b:explanation graphics, c: hybrid graphics). The use of data graphics (Fig. 17a) has increased dramatically. Statistically significant differences were observed between 1974 and 2014 (p < 0.001) (Table 5).

**Table 5:** Results of post hoc test of data graphics (p values)

	1974	1979	1984	1989	1994	1999	2004	2009	2014
1974									
1979	0.712								
1984	0.994	0.993							
1989	0.999	0.968	1.000						
1994	0.009**	0.591	0.116	0.061					
1999	0.320	1.000	0.861	0.729	0.918				
2004	0.000**	0.001**	0.000**	0.000**	0.275	0.007**			
2009	0.000**	0.000**	0.000**	0.000**	0.000**	0.000**	0.075		
2014	0.000**	0.000**	0.000**	0.000**	0.000**	0.000**	0.425	0.996	

^**^p < 0.01, *p < 0.05

The journal does not seem to have undertaken any explicit efforts to increase the number of figures in published content. The current web page of “Information for authors” contains the text "The cost for color figures is $1000 for the first color figure and $275 for each additional figure".^8^ It is not possible to keep track of the changes in the guidelines since the 2000s as they have been overwritten on the web pages, but at least in the 90 s and especially 2000s as well as the current guidelines require authors to pay for color figures. In addition, since 1993, it has been specified that the number of figures and tables should be kept within seven or eight. Despite these restraining instructions in the submission guidelines, the number of graphics has increased. There are a few possible reasons for this increase in data graphics.

**Fig. 18:**
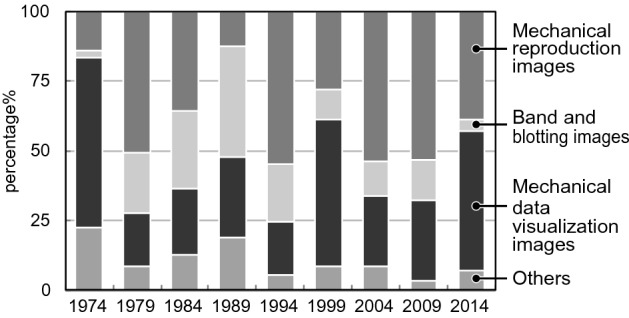
Details of data graphics

**Fig. 19:**
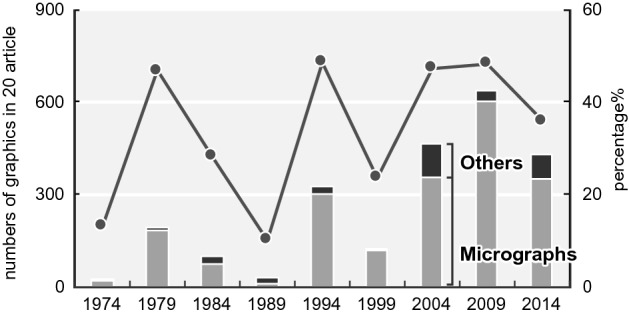
Changes in the numbers and ratio of mechanical reproduction images. The bar graph depicts real numbers and the line graph depicts percentages of for total graphics of each year

The first is the development of imaging and various data visualization technologies. Details of the data are presented in Fig. 18. The largest percentages are the mechanical reproduction images and mechanical data visualization images. Looking at the details of the mechanical reproduction images, the proportion of the micrographs is large (Fig. 19). We think that the spread of fluorescent microscopes using probes such as green fluorescent protein (GFP) from the late 1990s onwards has influenced this. The utility of GFP as a biological tool for gene expression was first reported in 1994 (Chalfie et al., 1994). This tool is one of the most important techniques in biology and medicine (Weiss, 2017). Moreover, the diversity of GFP variants has increased, and post-genome technologies such as multicolor imaging have been developed and have shown remarkable performance. When researchers use these technologies, they often use multicolor images (see the following figures for examples: Zhang et al., 2009; Fig. 5A–I; Asahina et al., 2014; Fig. 5) and time-lapse recording (see the following figures for examples: Linneweber et al., 2014, Fig. 5C and D; D’Angelo et al., 2009, Fig. 5B) which require many separate graphics. In our results, we encountered cases in which the authors had used more than 80 micrographs in a single article (see the following articles: Kim et al., 2009; Zhang et al., 2009). Because cases in which authors used a large number of images varied widely compared to those with fewer images, the ratio of mechanically reproduced images appears to have varied considerably over the years in our results. Other microscopic technologies have also been developed. Microscopic images have become clearer, and researchers have been able to perform more diverse analyses. Accordingly, researchers have engaged in scientific discussions that have utilized more images.

In addition, since the 2000s, the number of mechanical data visualization images has increased. In examining these images, there were several types of images for big data, such as network diagrams and heat maps (see the following figures for examples: Benton et al., 2009; Fig. 1; Kawahara et al., 2009; Fig. 6c (the lower part); Gräff et al., 2014, Fig. 5D). Visual methods, such as 3D structural analysis, network analysis, and big data analysis, have been developed and have become common in recent decades. Visualization technology based on computers is indispensable for researchers who need to study complicated and large amounts of data to grasp the meaning hidden in the data.

**Fig. 20:**
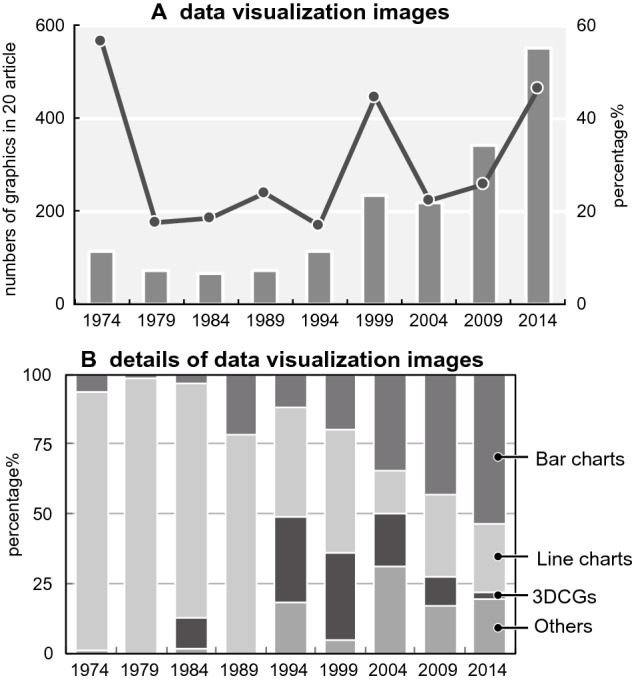
Changes of mechanical data visualization images. a: change in the numbers and ratio (the bar graph depicts real numbers and the line graph depicts percentages of for total graphics of each year). b: details

**Fig. 21:**
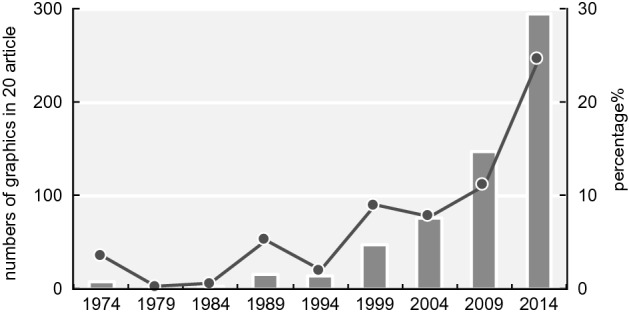
Changes in the numbers and ratio of bar charts. The bar graph depicts real numbers and the line graph depicts percentages of for total graphics of each year

The second possible reason for the increase in data graphics is that it has become easier to create graphics. The results of the mechanical data visualization images are presented in Fig. 20 and Table 6. The number of bar charts has increased dramatically (Fig. 21). There were statistically significant differences between 1974 and 2014 (p < 0.001) (Table 7). A previous study reviewing more than 700 original research articles in the top 25% of physiology journals found that 85.6% of the papers included at least one bar graph (Weissgerber et al., 2015). With the automation of experimental technologies, such as PCR and the efficiency of experimental methods, it has become possible to conduct more experiments in a shorter time. In addition, software for creating graphs and charts, such as Microsoft Excel, has become widespread. These changes have made it possible to quickly create more charts at low cost.

**Table 6:** Results of post hoc test of mechanical data visualization images (p values)

	1974	1979	1984	1989	1994	1999	2004	2009	2014
1974									
1979	0.999								
1984	0.998	1.000							
1989	0.999	1.000	1.000						
1994	1.000	0.999	0.998	0.999					
1999	0.587	0.198	0.160	0.211	0.587				
2004	0.745	0.317	0.265	0.335	0.745	1.000			
2009	0.011	0.001**	0.001**	0.001**	0.011*	0.745	0.587		
2014	0.000**	0.000**	0.000**	0.000**	0.000**	0.000**	0.000**	0.028*	

^**^p < 0.01, *p < 0.05

**Table 7:** Results of post hoc test of bar charts (p values)

	1974	1979	1984	1989	1994	1999	2004	2009	2014
1974									
1979	1.000								
1984	1.000	1.000							
1989	1.000	1.000	1.000						
1994	1.000	1.000	1.000	1.000					
1999	0.963	0.919	0.928	0.993	0.987				
2004	0.544	0.426	0.445	0.720	0.664	0.995			
2009	0.002**	0.001**	0.001**	0.006**	0.004**	0.096	0.504		
2014	0.000**	0.000**	0.000**	0.000**	0.000**	0.000**	0.000**	0.001**	

^**^p < 0.01, *p < 0.05

There are other reasons for the increase in bar charts. One is that a bar chart is one of the most familiar types of charts. Readers usually require literacy to read and interpret figures accurately (Trumbo, 2006). Using familiar expressions can reduce the risk of readers not being able to comprehend the data. This makes it easier to share an author’s interpretation with the readers. Bar charts can be used in a wider range of situations than line graphs because they can be used to represent non-contiguous data. Note that the number of line charts did not change as much as that of bar charts. Bar charts differ from line graphs in the following ways: (1) the horizontal axis of a bar chart consists of entities or processes rather than quantities (Gross & Harmon, 2014); (2) bar graphs tend to be used to convey discrete comparisons; and (3) line graphs tend to be used to convey trends (Zacks & Tversky, 1999). However, a previous study pointed out that univariate scatterplots, box plots, and histograms are more appropriate than bar and line charts for presenting continuous data with small sample sizes (Weissgerber et al., 2015).

A third possible reason motivating researchers to include more graphics is that providing data in a scientific context makes the manuscript more convincing and is associated with being objective. Data graphics are representations of numbers and images and are the basis for scientific claims. The increased number of data graphics indicates that research articles must be more convincing than before. There is also the argument that the researchers’ reaction is to win the acceptance of the journal. “Cell” is one of the most influential peer-reviewed scientific journals. It is recognized that getting accepted in top journals is valuable to researchers in promoting their careers. Brenner, the Nobel Laureate in Physiology or Medicine in 2002, criticized that publishing in high impact factor journals is a metric used to assess individual researchers, thus motivating researchers to produce research that caters to journals’ demands seeking sensational papers (Dzeng, 2014). Researchers have made great efforts to win acceptance by responding to the requests of editors and reviewers. Scientific data can be read differently than intended by the scientists who obtain the data and scientists often try to eliminate these alternative interpretations (Latour & Woolgar, 1979). In light of this, it is possible that researchers add data graphics in advance or in response to peer review in order to eliminate alternative interpretations of their data that reviewers (may) point out. Porter (1995) argued that quantification was related to reliability and grew in response to pressure from outsiders. According to the current web page on “Information for reviewers,”^9^ the core task of reviewers is to objectively assess both the technical rigor and the novelty of the work under review, and reviews can and should be critical. Although reviewers are usually members of the research community and not outsiders, they do not always agree with authors and could exert pressure on them. Adding images has become easier for researchers because more of the experimental technologies output images and the cost of creating images has decreased. Researchers may aim to convince reviewers with numerical data visualization, which seem "more real" (Latour, 1987) than the numbers from which the graphs are drawn. Hence, the observed increase in graphics may be associated with researchers' attempts to improve the validity of their articles’ claims.

**Fig. 22:**
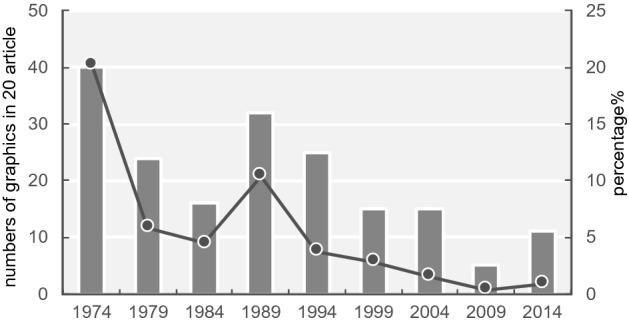
Change in the numbers and ratios of the tables. The bar graph depicts real numbers and the line graph depicts percentages of for total graphics of each year

The ratio of tables in the main bodies of the articles has decreased (Fig. 22). Statistically significant differences were observed between 1974 and 2009, with a p-value of less than 0.001 (Table 8). However, since 1999, tables have been included in both the main bodies and the supplemental information, and the number of tables in the supplementary information has increased (Tables 2 and 3). Although the number of tables as a whole has not changed significantly, data graphics have increased. This suggests that the data tend to be represented in other formats, such as graphs, rather than tables. Krohn (1991) showed that statistical graphs employ the powers of visual perception and pattern recognition to support the understanding of meaning. It is difficult to intuitively capture the entire image of the data from a table. There may not be much reason to actively select a table, rather than a graph. In the supplementary information, some tables have been provided in Excel format often exceeding the size that can be printed on one page in an article (see the following figures for examples: Déjardin & Kingston, 2009, Table S1; Asahina et al., 2014, Table S1; Chen et al., 2014, Table S2). The size and file format of the supplementary information are not as strict as those of the main body. The fact that some tables have been moved to supplementary information because of exceeding the size suggests that the limits of print have influenced the amount and presentation of data. Taylor and Blum (1991) discussed how representation has influenced printed features and graphic cultures. This results indicates that the features and trends in graphics differ between the print and online formats.

**Table 8:** Results of post hoc test of tables (p values)

	1974	1979	1984	1989	1994	1999	2004	2009	2014
1974									
1979	0.574								
1984	0.087	0.987							
1989	0.987	0.987	0.574						
1994	0.658	1.000	0.973	0.995					
1999	0.063	0.973	1.000	0.489	0.950				
2004	0.063	0.973	1.000	0.489	0.950	1.000			
2009	0.001**	0.332	0.915	0.031*	0.265	0.950	0.950		
2014	0.014*	0.808	1.000	0.207	0.737	1.000	1.000	0.998	

^**^p < 0.01, *p < 0.05

In the case of explanation graphics, there was no clear trend, such as that observed for data graphics (Fig. 17b). Explanation graphics are often used to explain experimental procedures, structures of experimental objects, interpretation of data graphics, and new models or theories (see the following figures for examples: Devare et al., 1984, Fig. 1; Johansson et al., 1999, Fig. 1a; Benton et al., 2009, Fig. 3A (graphics on the left); Cochran et al., 2009, Fig. 7).

**Fig. 23:**
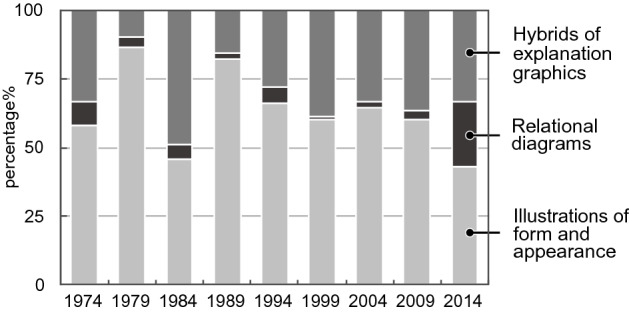
Details of explanation graphics

In the explanation graphics category, the group of illustrations of form and appearance accounted for a large percentage (Fig. 23). Although no clear trend emerged in the category of hybrids of explanation graphics, it was observed that they were used throughout the years considered (Table 2). However, their numbers were noticeably large in 1984. This was because of a single paper in 1984, in which 18 hybrids of explanation graphics appeared (see Miller et al., 1984). The influence was considered significant because the total number of participants was small.

When the hybrids of explanation graphics were examined in detail, it was found that they often represented theoretical models (see the following figures for examples: Drews et al., 1974; Fig. 1; Yuzhakov et al., 1999; Fig. 1; Fig. 5; Cochran et al., 2009; Fig. 7). These model diagrams include elements, such as molecules and cells, which are often connected by arrows. Specifically, the functions and relationships among the elements, such as movements, changes, and effects, are discussed using these diagrams. This is consistent with the fact that illustrations can effectively represent complex functions in science (Perini, 2005). Explanation graphics do not increase by large numbers because it is unlikely that a large number of experimental procedures and models will be presented in a single paper in the field of life sciences.

**Fig. 24:**
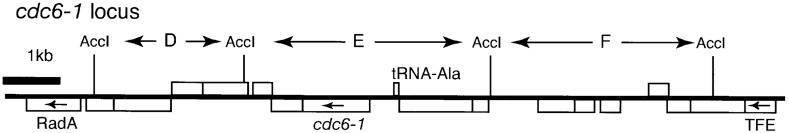
An example of a simple explanation graphic (Reprinted from Robinson et al., 2004, Fig. 1 with permission from Elsevier)

**Fig. 25:**
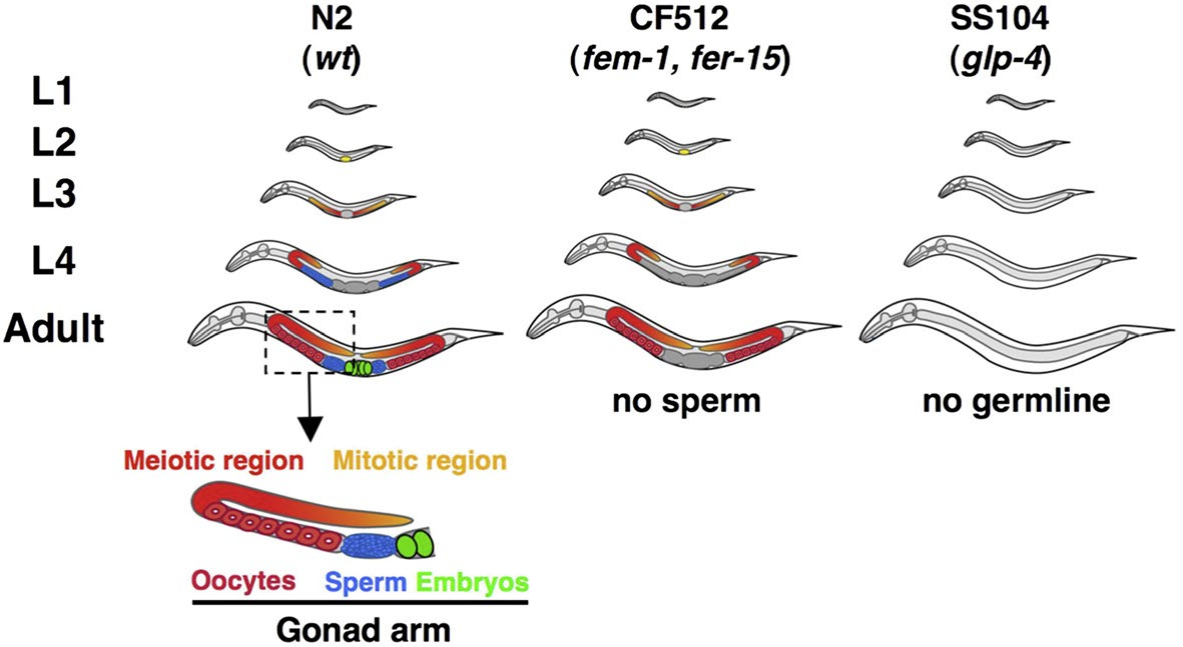
An example of a complex explanation graphic (Reprinted from D'Angelo et al., 2009, Fig. 2 with permission from Elsevier)

**Fig. 26:**
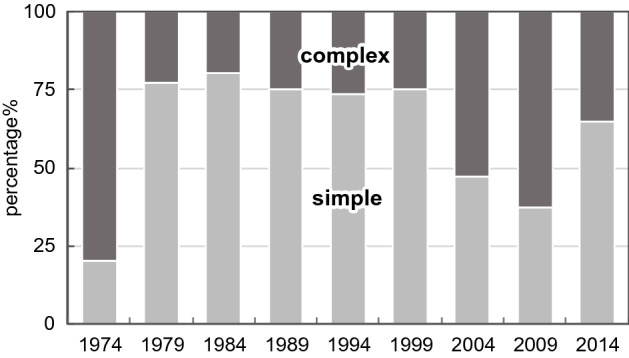
Change in the proportion of complex and simple explanation graphics

The representation of the illustrations was also analyzed. We defined a diagram that uses only letters, arrows, lines, and basic shapes (squares, rectangles, triangles, regular polygons, circles) was referred to as simple (Fig. 24) and a diagram that uses curves and cannot be expressed only by basic shapes was referred to as complex (Fig. 25). The results are shown in Fig. 26. Initially, complex diagrams appeared to be hand-painted (see the following figures for examples: Drews et al., 1974; Fig. 3; Periman & Hopper, 1979; Fig. 3; Zipursky et al., 1984; Fig. 2B), but in recent years, they have often been created using Bezier curves (see the following figures for examples: Johansson et al., 1999; Fig. 7; Tagami et al., 2004; Fig. 7; Kim et al., 2014; Fig. 7). Researchers typically create scientific illustrations using PowerPoint and Adobe Illustrator (Perkel, 2020). Compared to the complicated hand-drawn line drawings of the 70's, many model diagrams in recent years have been created by combining simple elements with arrows and lines, which are easy to create using PowerPoint and Adobe Illustrator. The ratio of “complex” diagrams has increased since the 2000s. We think this is because that the economic feasibility and usability of drawing applications have improved and that the drawing skills of researchers have also improved (see the following figures for examples: Saksena et al., 2009; Fig. 7; Snaidero et al., 2014; Fig. 7A–D; Gräff et al., 2014; Fig. 7). In the textbooks of biology, the diagrams tended to omit details and became more pedagogically sophisticated (Maienschein, 1991). In the reverse direction, diagrams in the journal have become also pedagogically sophisticated, evolving from simple diagrams with only basic shapes to diagrams that include complicated shapes that are intuitively easy to understand. Previous studies have shown that scientific illustrations are influenced not only by visual characteristics and the scientific knowledge of subjects but also by customs in arts and science, technologies, and historical background (e.g., Dance, 1978; Fleck, 1979; Ford, 1993). In addition to these factors, this study suggests that the form of application and scientists’ skills also influence the expression and meaning of scientific illustrations.

The number of hybrid graphics was too small to discuss variations over time although they have been used regardless of time (Table 2). In some cases, explanation graphics help interpret data graphics, and in other cases, data graphics are included as illustrations. Graphics are “shuffleable” and “recombinable” (Latour, 1990). Researchers can recombine data and explanation graphics more easily using applications such as Microsoft PowerPoint, Keynote, Adobe Photoshop, and Adobe Illustrator. Such fusion and diversification blur the boundaries of the role between data graphics and illustrations. The imaging software can influence how visual arguments are presented in science.

## Conclusion

In this study, the change in scientific imaging trends was examined since the spread of computer-based visual and imaging technology. To do this, a new classification method was constructed for journal figures to enable the analysis of trends in the figures. The main categories observed were (A) data graphics, (B) explanation graphics, and (C) hybrid graphics. We found that data graphics have diversified and increased in number over time, from 183 (1974) to 1196 (2009) in 20 articles. We suggest that this change is related to the development of experimental technologies, the spread of image creation software, and researchers’ incentives to convince reviewers and editors. We also point out that the method of creating graphics has changed. The spread of software, such as PowerPoint and Adobe Illustrator, and the unification of image data have brought about changes in the trend of scientific graphics. It is also demonstrated that graphics features and trends differ between print and online formats.

This study has some limitations. First, classification was performed manually rather than automatically. There is a limit to the number of graphics that can be analyzed although compared with previous approaches that analyzed at most dozens of images, several thousands of images were analyzed in this study. The study has an intermediate role between qualitative and quantitative approaches.

Second, graphics may be classified in various ways. In this study, we considered three main categories. The process of constructing a classification using the KJ method includes intuition (Scupin, 1997). Therefore, the appropriate taxonomy may differ between applications, and various classification methods may be suitable depending on the intended objective.

Third, there may be a large difference in the usage trends of figures in each scientific field. The journal *Cell*, as the name suggests, covers various fields related to cells. Considering the differences in convention between fields, some of the results might be considered artifacts resulting from crossing between these specializations. On the other hand, because modern life sciences include various fields, and the specific classification of the fields may depend on research methods, approaches, interests, and species, it is difficult to identify the specific scientific field of the manuscript. Thus, it is more effective to focus on one topic or a specific author for analysis (for example, the approach of Bechtel et al., 2014; Burnston et al., 2014). On the other hand, with such an approach, it is difficult to grasp the big picture in terms of the usage of figures in one field, and the influence of the individual researcher may also be strong. We attempted to see a major trend in the scientific journal *Cell*, which incorporates multiple fields related to cell research.

In the future, to understand trends in the use of scientific graphics, large-scale analyses will be conducted using artificial intelligence (AI) techniques. In addition, more detailed discussions can be carried out by combining the results of this study with an analysis of qualitative data, such as interviews with scientists. The results of this research are considered useful not only for historical studies of science but also for the education of researchers and in the discussion of scientific misconduct.

## Data Availability

None.

## Footnotes

1 In this paper, a figure labeled by the journal (for example, “Fig. 1” and “Fig. 2”) is referred to as a "Figure” in order to distinguish it from a figure in the sense of a general drawing or image. Similarly, a table labeled by the journal is referred to as a "Table".

2 Cell Press “Aims and Scope” https://www.cell.com/cell/home. Accessed 2 April 2022.

3 The KJ method (or affinity diagrams) is a method for organizing ideas and data. It was originally developed by Jiro Kawakita, a Japanese anthropologist (Kawakita, 1986), and is has been utilized globally ever since. It helps to organize fragmented information into logical, cohesive groups and generate a limited number of categories.

4 The reason for collecting the five articles every five years in the preliminary classification is that it was the limit amount that could grasp the whole picture of the graphics using the KJ method. Even if we overcome this cognitive problem and perform steps (1)–(4) and (5) at once, the results are considered to be the same.

5 Elsevier “Graphical abstracts” https://www.elsevier.com/authors/tools-and-resources/visual-abstract Accessed 5 August 2021.

6 According to the supplemental information guidelines of *Cell*, supplemental information is “data and other materials that directly support the main conclusions of a paper but are considered additional or secondary support for the main conclusions or cannot be included in the main paper for reasons such as space or restrictions on file format.” It is considered that the functions and usage trends of graphics may differ between the main text bodies and supplemental information. However, as far as we know, it is not clear how different they are. In the Sects. 3.3 and 3.4, we primarily discussed the results of graphics of the main bodies of the articles and presented the result of supplementary information in Table 3 only as a supplement of our discussion.

7 In this paper, a bar graph shows a change in total item’s numbers in a specific category of 20 articles per year, and a line graph shows a change in ratios (%) of items in a specific category to the numbers of all graphics in 20 articles per year.

8 Cell Press “Instructions for authors” https://www.cell.com/cell/authors Accessed 2 April 2022.

9 Cell Press “Information for reviewers” https://www.cell.com/reviewers Accessed 2 April 2022.
